# Investigation of a tellurium-packed column for isolation of astatine-211 from irradiated bismuth targets and demonstration of a semi-automated system

**DOI:** 10.1038/s41598-019-53385-x

**Published:** 2019-11-18

**Authors:** Yawen Li, Donald K. Hamlin, Ming-Kuan Chyan, Taylor M. Morscheck, Maryline G. Ferrier, Roger Wong, D. Scott Wilbur

**Affiliations:** Department of Radiation Oncology, University of Washington, Seattle, Washington United States of America

**Keywords:** Molecular medicine, Nuclear chemistry

## Abstract

Astatine-211 is an attractive radionuclide for use in targeted alpha therapy of blood-borne diseases and micrometastatic diseases. Efficient isolation methods that can be adapted to robust automated ^211^At isolation systems are of high interest for improving the availability of ^211^At. Based on the early studies of Bochvarova and co-workers involving isolation of ^211^At from irradiated thorium targets, we developed a method for ^211^At isolation from bismuth targets using tellurium-packed columns. Dissolution of irradiated bismuth targets is accomplished using HNO_3_; however, ^211^At is not captured on the Te column material in this matrix. Our method involves slow addition of aqueous NH_2_OH·HCl to the Bi target dissolved in HNO_3_ to convert to a HCl matrix. The amount of NH_2_OH·HCl was optimized because (1) the quantity of NH_2_OH·HCl used appears to affect the radiolabeling yield of phenethyl-*closo*-decaborate(2-) (B10)-conjugated antibodies and (2) reducing the volume of NH_2_OH·HCl solution can effectively shorten the overall isolation time. A proof-of-concept semi-automated process has been demonstrated using targets containing ~0.96 GBq (~26 mCi) of ^211^At. High isolation yields (88–95%) were obtained. Radiochemical purity of the isolated ^211^At was assessed by radio-HPLC. Concentrations of Bi and Te contaminants in the ^211^At and the astatinated antibodies were evaluated using ICP-MS.

## Introduction

Astatine-211 is one of the most attractive radionuclides for targeted alpha therapy (TAT)^1–4^. It has a 7.21 h half-life, very low abundance of high energy gamma-ray emissions and 100% alpha emission. Astatine-211 can be produced by bombardment of high purity bismuth metal (naturally monoisotopic) targets with 28–29 MeV alpha particles. Dry distillation is widely used for isolation of ^211^At from irradiated Bi targets. Various dry distillation set-ups have been described in the literature^5–11^, but handling of radioactive gases has raised concerns by institutional radiation safety officials. Moreover, if large quantities of ^211^At are to be produced, the size of bismuth target must be increased significantly. This increase in size of target is due to the low melting point of Bi (271.5 °C), requiring that the alpha beam be spread over a large area to provide efficient cooling during irradiation. Implementing the dry distillation method when using larger bismuth targets can present significant challenges due to the fact that increasing the size of the quartz tube for distillation of Bi can make it difficult to find commercial tube ovens for this purpose and the larger tubes can make the distillation process less efficient^12^. An alternative to placing large irradiated bismuth targets (and their backing material) in an oven is to mechanically remove the irradiated bismuth from the target backing, followed by placing the bismuth in a high temperature oven (650–750 °C)^5^. The safety of this process has also raised concerns from radiation safety officials.

An alternative method of isolating ^211^At from irradiated bismuth targets involves “wet chemistry” liquid-liquid extraction processes^13–17^. We have been able to consistently obtain high isolation yields using this method, but it is a 2.5–3 hour process where distillation of concentration HNO_3_ and multiple liquid-liquid extraction steps are required^12^. In the isolation process irradiated bismuth targets are dissolved in concentrated HNO_3_, but the organic phase used, diisopropyl ether (DIPE), cannot effectively extract ^211^At from HNO_3_ solutions. Therefore, the HNO_3_ is removed by distillation and 8 M HCl is used to re-dissolve the Bi(NO_3_)_3_ salt residue. Liquid-liquid extractions are then performed to isolate ^211^At from the ^211^At/Bi^3+^ mixture and to remove Bi^3+^ salts using DIPE and 8 M HCl. We have investigated automation of this wet chemistry, liquid-liquid exaction method for ^211^At isolation, and although it has been technically challenging, we have had some success in automation^18^. Unfortunately, thus far we have not been able to decrease the time required using the automated “wet chemistry” ^211^At isolation process from that achieved in the manual separation procedure (unpublished data). In an effort to simplify the isolation process and decrease the time to obtain the isolated ^211^At, we looked for alternative isolation methods.

During the separation process in the wet chemistry method, astatine undergoes several changes in its oxidation state, possibly from At(+5) to At(+3), then to At(0), and finally to At(−1)^14^. Along with the change in oxidation state there are likely different chemical species produced. In addition to astatide, four other unknown astatine species have been observed at different times by anion exchange radio-HPLC^12^. The inconsistency in the radiochemical purity of the ^211^At isolated using the DIPE extraction method can result in poor radiolabeling yields, which can be a major problem in fulfilling prescribed doses in the clinical setting.

Here we report a new ^211^At-isolation approach based on a tellurium-packed column (Te column) previously described in the literature. Bochvarova *et al*. reported a method of using two tellurium metal packed columns to effectively isolate ^211^At from 660 MeV proton beam irradiated thorium targets^19^. Astatine-211 can be rapidly absorbed on metallic tellurium in HCl in the presence of SnCl_2_ and eluted by a solution of 1–2 M NaOH. In order to adapt the Te column method to irradiated bismuth targets, we used NH_2_OH⋅HCl to convert the HNO_3_ solution containing the dissolved target to a solution of HCl. We also demonstrated this new method can be readily automated and can provide [^211^At]NaAt of consistent and high radiochemical purity.

## Results

### At-211 isolation using Te columns

**Figure 1:**
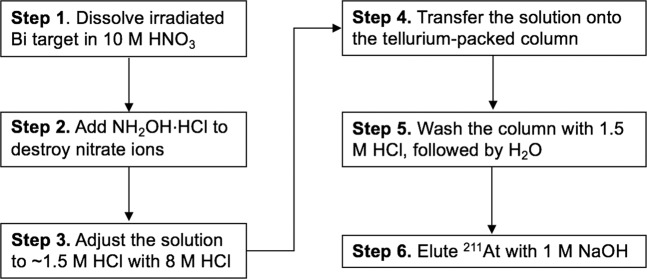
Flow chart for the isolation process of ^211^At from irradiated Bi targets using Te columns.

Figure 1 summarizes the steps involved in the process of isolating ^211^At from irradiated Bi targets using Te columns. Step 1 involves dissolution of the bismuth target using 10 M HNO_3_. This is the same initial step used in the automation of the wet chemistry isolation process^18^. Step 2 involves addition of NH_2_OH·HCl to the HNO_3_ solution containing dissolved Bi dropwise until complete cessation of bubbling is noted. Steps 3–6 are conducted as shown in Fig. 1. However, in our initial studies steps 3 and 5 were slightly different, as a reductant, SnCl_2_ was used to assure that the ^211^At was in the astatide form. The steps initially used were as follows: In step 3, 0.1 M SnCl_2_ in 6 M HCl was used instead of 1.5 M HCl and in step 5, the column was washed by 0.1 M SnCl_2_ in 6 M HCl, 6 M HCl and deionized (D.I.) H_2_O sequentially. As NH_2_OH·HCl is a strong reducing agent, the use of 0.1 M SnCl_2_ seemed to be redundant but we thought it would be best to evaluate isolation yields with and without having SnCl_2_ present.

Table 1 shows that the astatine adsorption kinetics of the Te column is very fast and efficient, irrespective of whether SnCl_2_ was used or not. Essentially all of the ^211^At was absorbed by the Te column from 1.5 M HCl even at a high flow rate of 5–10 mL/min. Moreover, very little activity was found in the washes (<0.1%) and isolation yields ~75% were obtained when the ^211^At/Bi mixture was adjusted to 0.1 M SnCl_2_ in 6 M HCl. Comparable or even higher ^211^At isolation yields were obtained without the use of SnCl_2_ (Table 1). Therefore, the later ^211^At isolation experiments were conducted without SnCl_2_, as outlined in Fig. 1.

**Table 1:** Isolation yields of ^211^At using Te columns in the presence or absence of SnCl_2_ in the loading mixture^*a*^.

Loading solution	% Captured by Te column	% Activity in HCl wash (20 mL)	% Activity in H_2_O wash (20 mL)	%^211^At eluted in 1st 1 mL NaOH (decay-corrected)
1.5 M HCl	>99.9	<0.1	<0.1	78.9–87.7
0.1 M SnCl_2_ in 6 M HCl	99.5	<0.1^*b*^	<0.1	75

^*a*^35% aqueous NH_2_OH⋅HCl was added until the cessation of bubbling. ^*b*^The Te column was eluted with 20 mL of 0.1 M SnCl_2_ in 6 M HCl, followed by 20 mL of 6 M HCl.

### Semi-automated ^211^At isolation runs

**Figure 2:**
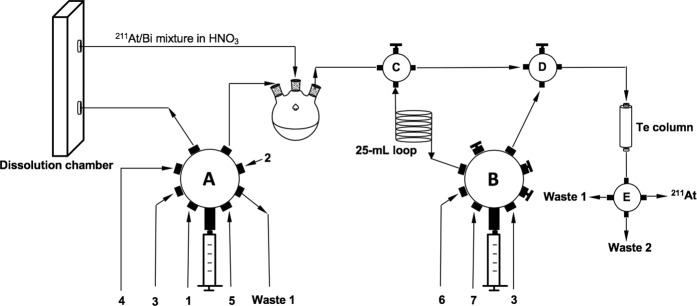
Schematic of the semi-automated process of ^211^At isolation using a Te column. In the process two syringe pumps move the solvents and mixtures through valves (**A–E**). Numbered solutions are as follows: **1**: 10 M HNO_3_; **2**: air; **3**: D.I. H_2_O; **4**: 35% NH_2_OH⋅HCl; **5**: 8 M HCl; **6**: 1.5 M HCl; **7**: 1 M NaOH. **Waste 1**: non-radioactive waste; **Waste 2**: radioactive waste. (Detailed description of equipment and reagents can be found in Fig. S1).

The results of the manual ^211^At isolation runs were very encouraging, so the procedure was adapted to a semi-automated system to demonstrate potential application of the Te column method for routine ^211^At isolation. Three proof-of-concept semi-automated ^211^At isolation runs were conducted using irradiated bismuth targets following the procedure outlined in Fig. 1. The schematic of the semi-automated process is shown in Fig. 2. A picture of our prototype is shown in Fig. S1. Results of the semi-automated runs are summarized in Table 2. The irradiated bismuth targets each contained about 962 MBq (26 mCi) and the overall ^211^At isolation process run time was 90–100 minutes. On average, the system was able to recover 93±4% of the ^211^At in 2 mL of 1 M NaOH. For the 1^st^ and 2^nd^ semi-automated runs, 80 mL of 35% aqueous NH_2_OH⋅HCl was used and the columns were washed with 20 mL of 1.5 M HCl, followed by 20 mL of deionized (D.I.) H_2_O. For the 3^rd^ semi-automated run, the volume of 35% NH_2_OH⋅HCl was reduced by 15 mL which appears to be enough to destroy all of the nitrate. Decreasing the volume of aqueous NH_2_OH⋅HCl reduced the overall run time, even though an additional 20 mL of 1.5 M HCl and D.I. H_2_O was used for washing the column in the 3^rd^ semi-automated run.

**Table 2:** Quantities of reagents, isolated yields and run times from three semi-automated ^211^At isolation experiments.

	NH_2_OH·HCl (mL)	1.5 M HCl wash (mL)	H_2_O wash (mL)	% Isolation yield (decay-corrected)	Run time (min)
1	80	20	20	95.3	100
2	80	20	20	88^*a*^	100
3	65	40	40	96.7	90

^*a*^Lost 5–10% of the activity because a ferrule fitting of the dissolution chamber leaked during the run.

### Optimization of the amount of NH_2_OH·HCl

Although the ^211^At isolation yields were encouraging when an excess of NH_2_OH·HCl was used, we obtained inconsistent radiolabeling yields, ranging from 10.4% to 94.7%, when using the isolated ^211^At solutions to label isothiocyanato-phenethyl-*closo*-decaborate (2-) (B10-NCS)-conjugated monoclonal antibodies (MAbs) at room temperature in the absence of an oxidant. We also observed that addition of the oxidant chloramine-T improved the MAb-B10 radiolabeling yield, suggesting that there might be reductive impurities in the isolated ^211^At solution.

We hypothesized optimizing the amount of NH_2_OH·HCl might mitigate this problem. Experiments were conducted to determine the minimal volume of 35% NH_2_OH·HCl required to destroy all the NO_3_^−^ (V_min_) in the HNO_3_ solution containing the dissolved Bi target, as it seemed to be impossible to accurately assign defined stoichiometry to the reaction between NH_2_OH·HCl and HNO_3_. Minimally, about 52 mL of 35% NH_2_OH·HCl is needed for a Bi target that is dissolved in 15 mL of 10 M HNO_3_. A series of manual ^211^At isolations were conducted using different percentages of V_min_. Table 3 shows that although the ^211^At isolation yield decreased as the volume of 35% NH_2_OH·HCl was reduced, the radiolabeling yields for B10-conjugated MAb increased significantly. Reducing the amount of NH_2_OH·HCl to 52% of V_min_ appears to be optimal, as the radiolabeling yield increased to 82.5%, equivalent to the normal radiolabeling yield achieved with ^211^At isolated using the DIPE method. And at 52% of V_min_, an isolation yield of about 80% could still be obtained.

**Table 3:** Using various amounts of NH_2_OH⋅HCl for ^211^At isolation and its influence on the B10-conjugated MAb labeling yield.

NH_2_OH·HCl (% V_min_)	% Captured by Te column	% Activity in HCl wash (20 mL)	% Activity in H_2_O wash (20 mL)	% ^211^At eluted in the 1st 1 mL NaOH (decay-corrected)	% B10-conjugated MAb labeling yield
≥100	>99.9	<0.1	<0.1	78.9–87.7	10.4–94.7
95	>99.9	<0.1	<0.1	86.6	17.0
83	>99.9	<0.1	<0.1	83.7	26.0
80	98.7	<0.1	<0.1	80.5	23.4
68	>99.9	0.13	<0.1	78.1	29.8
52	99.5	0.20	<0.1	79.8	82.5
46	98.9	0.30	<0.1	70.2	72.9

### Chemical and radiochemical purity

The Te column method can effectively isolate ^211^At from large-sized Bi targets containing 4–5 g of Bi metal, but trace levels of Te and Bi impurities in the isolated ^211^At solutions were anticipated. The concentrations of Te and Bi impurities in the ^211^At solutions were evaluated by ICP-MS after the ^211^At decayed. Calibration curves were generated using Bi and Te standard solutions at concentrations of 1, 10, 50 100 ppb, with R^2^ ≥0.9995. Recoveries of the internal standards (ISTDs) were very close to 100% for all samples including calibration standards. On average, the concentration of residual Bi in the isolated ^211^At solution was 3.0 ppm, which is slightly higher than that of the ^211^At isolated using the DIPE method (Table 4). The concentration of the Te contaminant in the isolated ^211^At solution is rather high, about 32.8 ppm on average. However, it should be noted that ^211^At-labeled MAb prepared from Te column isolated ^211^At and purified by a size-exclusion (PD-10) column had significantly reduced Bi and Te concentrations of about 0.05 and 0.04 ppm, respectively (Table 4).

**Table 4:** Levels of Te and Bi impurities in isolated ^211^At and ^211^At-labeled MAb.

	Te (ppm)	Bi (ppm)	
	Te column	Te column	DIPE
[^211^At]NaAt (n = 10)	32.8 ± 15.8	3.0 ± 2.5	1.7 ± 1.5
^211^At labeled MAb (n = 3)	0.04 ± 0.01	0.05 ± 0.04	n.a.

**Figure 3:**
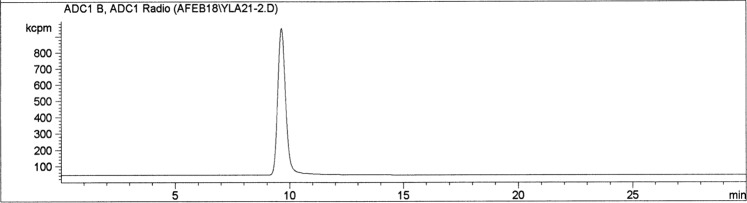
Representative anion exchange radio-HPLC chromatogram of the [^211^At]NaAt isolated using the Te column method.

Anion exchange radio-HPLC analyses were performed to evaluate the radiochemical purity of the isolated ^211^At. A representative radio-chromatogram is shown in Fig. 3. Only one radiopeak at 9.5 min has been observed for several ^211^At solutions purified using the Te column method (n > 10), which suggests radiochemical purity >99% can be consistently achieved using the Te column method.

## Discussion

Astatine-211 can be rapidly absorbed on metallic Te in HCl in the presence of SnCl_2_ and eluted by a solution of 1–2 M NaOH. The high affinity of At^−^ to elemental Te in HCl might be the result of the formation of a coordination bond between the surface Te and the highly polarizable At^− ^^19^. In the literature, Te columns were used for separation of ^211^At from irradiated thorium targets^19^ or polonium impurities, when ^211^At is produced via high energy proton induced spallation of thorium^19^ or the ^209^Bi(^7^Li, 5n)^211^Rn → ^211^At route^20^, respectively. In those scenarios, the solutions containing ^211^At/impurities do not contain large amounts of HNO_3_ and can be readily diluted and constituted to approximately 0.1 M SnCl_2_ in 6 M HCl prior to loading onto the Te column. However, dissolution of our Bi targets which contain 4–5 grams of Bi metal requires the use of 15–17 mL of 10 M or concentrated HNO_3_. Hydroxylamine can reduce HNO_3_ to HNO_2_ which further reacts with NH_3_OH^+^ and produce gaseous N_2_O (g), and N_2_ (g), so it is used to convert the nitrate matrix to HCl^21,22^. The addition of NH_2_OH⋅HCl also eliminates the need for using SnCl_2_ in the solution transferred onto the Te column. It is likely that NH_2_OH⋅HCl reduces astatine in other oxidation states to astatide, the astatine species that might be required for the Te column method to work properly.

Compared to the DIPE extraction method, using NH_2_OH⋅HCl for converting the HNO_3_ solution containing the dissolved Bi target to a HCl matrix is not only easier to automate, but can be faster than distilling the HNO_3_ to dryness. It takes ~25 min to completely destroy the nitrate, adding the NH_2_OH⋅HCl solution (V_min_ = 52 mL) using the semi-automated system, which is comparable to the time it takes to remove the HNO_3_ by distillation (~30 min)^12^. However, we found that not all of the nitrate needs to be destroyed to obtain good isolation yields. In fact, adding 52% of V_min_ provides high ^211^At isolation yields as well as high B10-conjugated MAb labeling yields (Table 3).

The overall run times of the semi-automated ^211^At isolation experiments are 20–30 min shorter than those of the DIPE extraction process (Table 2). The high affinity of At^−^ to elemental Te allows adsorption of ^211^At onto Te columns and washing the Te columns with HCl and H_2_O at high flow rates of 6 mL/min. Also, the elution of ^211^At from the Te column using NaOH is very efficient, averaging 93% of the ^211^At in 2 mL volume at a flow rate of 60 mL/min. It should be noted the NaOH back extraction step in the DIPE extraction method can takes 10–20 min to finish^12^. The fast flow rates used in the Te column isolation process are critical for achieving good ^211^At isolation yields in a reasonable amount of time, especially, considering the volume of ^211^At solution passing over the Te columns is rather large.

Astatine-211 solutions obtained using the DIPE liquid-liquid extraction method can have multiple astatine species which can lead to low radiochemical purity^12^. In contrast, ^211^At solutions isolated using Te columns consistently provide only astatide in a radiochemical purity >99%. This might be due to ^211^At being reduced to astatide by NH_2_OH⋅HCl before being transferred onto the column. However, it must be noted that a small amount of tellurium metal is dissolved in 1 M NaOH as ^211^At is eluted off the column and Na_2_TeO_3_ is a weak reducing agent. While untested at this time, the presence of Na_2_TeO_3_ might cause problems for astatine labeling, especially electrophilic astatination reactions. Thus, methods for purifying ^211^At from the Te (and possibly Bi) impurities need to be evaluated for applications that require higher purity.

In conclusion, Te columns provide an alternative method for efficiently isolating ^211^At from irradiated Bi targets. The isolated ^211^At solution is of high radiochemical purity and is suitable for B10-conjugated MAb labeling. A semi-automated process based on the Te column method has been demonstrated. Studies to evaluate the influence of the mesh size of the Te powder used on the ^211^At isolation yield are on-going. As future work, the geometry of the Te column, the volumes of reagents including 1.5 M HCl, D.I. H_2_O and 1 M NaOH need to be optimized to minimize the overall run time and to reduce the volume of the final product to 0.5 mL or less.

## Methods

### Reagents and general procedures

The chemicals and reagents used were purchased from VWR International (Radnor, PA), Sigma Aldrich (St. Louis, MO) or Fisher Scientific (Pittsburgh, PA), and were used without further purification unless otherwise specified. Empty Mini Spe-ed column cartridges were obtained from Applied Separations (Allentown, PA). ICP-MS tuning solution and 10 μg/mL standard solutions of Bi and Te were obtained from Inorganic Ventures (Christiansburg, VA). Monoclonal antibodies were obtained from the Fred Hutchinson Cancer Research Center Biologics Production Facility and isothiocyanato-phenethyl-*closo*-decaborate (2-) (B10-NCS)-conjugated MAbs were prepared in house as previously described^23^. Astatine-211 was produced by irradiation of Bi metal on an aluminum target support with 29 MeV α-particles using the Scanditronix MC50 cyclotron as previously described^24^. Various solutions containing ^211^At generated before and after Te column separation were measured in a Capintec CRC-55tR dose calibrator using the calibration setting number 44. Astatination of the B10-conjugated MAbs with ^211^At isolated using Te columns were conducted as previously described^25^.

### Determine the minimal volume of NH_2_OH·HCl

Determining the production rate of each of the products generated by the HNO_3_ and NH_2_OH·HCl redox reaction was not attempted. It seemed that accurately assigning a defined stoichiometry to this reaction was not possible, thus the minimal volume of 35% NH_2_OH·HCl required to destroy all the nitrate ions in the dissolved Bi target solution (V_min_) was determined experimentally.

To mimic the semi-automated isolation process, 4.25 g of Bi metal was dissolved in 15 mL of 10 M HNO_3_. The resultant solution was split into three 5-mL fractions and 35% aqueous NH_2_OH·HCl was added dropwise at a flow rate of approximately 2 mL/min using a 25-mL burette. The completion of the NH_2_OH·HCl and nitric acid reaction was observed as a cessation of bubbling. Nitrate/nitrite test paper (EMD Millipore^TM^) was used to verify the nitrate ion was below the detection limit (10 ppm).

### Manual ^211^At isolation using Te columns

Each Mini Spe-ed column cartridge (Applied Separations, Allentown, PA) was filled with 1 g (dry weight) of tellurium metal powder (−200 mesh). The columns were washed sequentially by 20 mL of 2 M NaOH, 3 M HCl and D.I. H_2_O. Prior to use, the Te columns were pre-equilibrated with 20 mL of 0.1 M SnCl_2_ in 6 M HCl or 1.5 M HCl.

An irradiated Bi target containing ~0.96 GBq (~26 mCi) of ^211^At was placed Bi face down in a plastic container. A total of 17 mL of concentrated HNO_3_ was manually added to dissolve the Bi and ^211^At. Because 10 M HNO_3_ rather than concentrated HNO_3_ would be used for target dissolution in the semi-automated ^211^At isolation process, 1 g of high purity (99.999% trace metal basis) Bi beads were dissolved in 3 mL of 10 M nitric acid to mimic the Bi^3+^ and NO_3_^−^ concentration in the dissolved Bi target solution obtained using the semi-automated process. The resultant solution was spiked with 0.5–1 mL concentrated HNO_3_ of dissolved irradiated Bi targets. Depending on the chromatographic conditions to be evaluated, the obtained solution was adjusted accordingly.

*Loading in 0.1 M SnCl*_2_ *in 6 M HCl*. Thirty five percent NH_2_OH·HCl was added dropwise until the cessation of bubbling. The solution was then adjusted to 0.1 M SnCl_2_ in 6 M HCl using 1 M SnCl_2_ and concentrated HCl. After loading the resultant solution onto the Te column, the column was eluted with 20 mL of 0.1 M SnCl_2_ in 6 M HCl, followed by 20 mL of 6 M HCl, D.I. H_2_O and 2 mL of 1–2 M NaOH.

*Loading in 1.5 M HCl*. Various amounts of 35% aqueous NH_2_OH·HCl were added dropwise, followed by 3–4.35 mL of 8 M HCl to reconstitute the solution to approximately 1.5 M HCl. After loading the resultant solution onto the Te column, the column was eluted by 20 mL of 1.5 M HCl, D.I. H_2_O and then 2 mL of 1–2 M NaOH.

### The semi-automated ^211^At isolation process

The schematic of the semi-automated system is shown in Fig. 2 and component details of the system are provided in Fig. S1. An irradiated Bi target containing ~0.96 GBq (~26 mCi) of ^211^At was manually put into the polyetherimide (ULTEM^TM^) dissolution chamber^18^, then the semi-automated ^211^At isolation process was started from the computer. The magnetic stirrer was turned on manually. Using the 10-mL syringe installed on the Hamilton syringe pump **A**, 15 mL of 10 M HNO_3_ (**1**) was pumped through the dissolution chamber to dissolve the target at a flow rate of 2.4 mL/min. The HNO_3_ solution obtained from the dissolved target was collected in the 250-mL round bottom flask. Air (**2**) was pushed through the dissolution chamber to ensure all the HNO_3_ was transferred into the round bottom flask. D. I. H_2_O (**3**) was used to rinse the 10-mL syringe and was moved into Waste 1. Using the same 10-mL syringe, at a flow rate of 2.4 mL/min, 65 mL or 80 mL of 35% NH_2_OH·HCl (**4**) was added to the dissolved target solution to destroy the nitrate ions, followed by 17.1 mL or 22 mL of 8 M HCl (**5**) to make the solution 1.5 M in HCl. The total volume after these steps was about 97 mL or 117 mL, depending on volume of 35% NH_2_OH·HCl used. Using a 25-mL syringe on Hamilton syringe pump **B**, 25 mL of 1.5 M HCl (**6**) was passed over the Te column into Waste 1 to pre-equilibrate the column. After the column was equilibrated, 21.5 mL of the dissolved target solution was transferred into a 25-mL loop, then flowed through the Te column at a flow rate of 6 mL/min. This step was repeated multiple times to load all the radioactive solution onto the column. During this process, the effluent from the column containing Bi and other impurities was delivered into Waste 2. The Te column was then rinsed with 20 or 40 mL of 1.5 M HCl, followed by 20 or 40 mL of D.I. H_2_O. Finally, the column was eluted using 5–7 mL of 2 M NaOH in 1 mL fractions at a flow rate of 60 mL/min.

### Radio-HPLC and ICP-MS analysis

*Radio-HPLC*. Radio-HPLC analyses were performed on the ^211^At solutions using a Hewlett-Packard model 1050 HPLC (Hewlett-Packard Company, Palo Alto, CA) with a Beckman Model 170 Radioisotope Detector (Beckman-Coulter, Brea, CA). Isocratic analyses were conducted on a Dionex IonPac AS-20 anion exchange column with a Dionex AG-20 guard column (Dionex, Sunnyvale, CA), eluting with a 50 mM NaOH solution at 1.3 mL/min.

*ICP-MS*. Determination of the concentrations of Bi and Te impurities in the isolated ^211^At product was carried out using an Agilent 7900 ICP-MS and the associated Mass Hunter Workstation software package. High purity HNO_3_ and ultrapure H_2_O (≥17.8 MΩ·cm) were used to prepare ICP-MS samples. Ten μL aliquots of ^211^At solutions were diluted 1,000 times in 5% HNO_3_. Samples containing antibodies were heated for 1 h in concentrated high purity HNO_3_ at 99 °C before diluting to 5% HNO_3_. A 100-ppb solution of Co, Y, Ce, and Tl was used as internal standard (ISTD). The ISTD was aspirated by a separate line and mixed with samples or standard solutions before the nebulizer using an online ISTD addition connector.

## Supplementary information

Investigation of a tellurium-packed column for isolation of astatine-211 from irradiated bismuth targets and demonstration of a semi-automated system

## Data Availability

All data generated and analyzed during this study are included in this article and are also available from the authors upon request.

## References

[1] Chen Y (2012). Durable donor engraftment after radioimmunotherapy using α-emitter astatine-211-labeled anti-CD45 antibody for conditioning in allogeneic hematopoietic cell transplantation. Blood.

[2] Green DJ (2015). Astatine-211 conjugated to an anti-CD20 monoclonal antibody eradicates disseminated B-cell lymphoma in a mouse model. Blood.

[3] Zalutsky MR, Reardon DA, Pozzi OR, Vaidyanathan G, Bigner DD (2007). Targeted α-particle radiotherapy with ^211^At-labeled monoclonal antibodies. Nuclear Medicine and Biology.

[4] Baidoo KE, Yong K, Brechbiel MW (2013). Molecular Pathways: Targeted α-Particle Radiation Therapy. Clin Cancer Res.

[5] Aneheim E, Jensen H, Albertsson P, Lindegren S (2015). Astatine-211 labeling: a study towards automatic production of astatinated antibodies. J Radioanal Nucl Chem.

[6] Hadley SW, Wilbur DS, Gray MA, Atcher RW (1991). Astatine-211 labeling of an antimelanoma antibody and its Fab fragment using N-succinimidyl p-[^211^At]astatobenzoate: comparisons *in vivo* with the p-[^125^I]iodobenzoyl conjugate. Bioconjugate Chemistry.

[7] Koziorowski J, Lebeda O, Weinreich R (1999). A cryotrap as flow reactor for synthesis of ^211^At labelled compounds. Applied Radiation and Isotopes.

[8] Lindegren S, Bäck T, Jensen HJ (2001). Dry-distillation of astatine-211 from irradiated bismuth targets: a time-saving procedure with high recovery yields. Applied Radiation and Isotopes.

[9] Wilbur DS (1993). Preparation and evaluation of para-[^211^At]astatobenzoyl labeled anti-renal cell carcinoma antibody A6H F(ab′)_2_. *In vivo* distribution comparison with para-[^125^I]iodobenzoyl labeled A6H F(ab′)_2_. Nuclear Medicine and Biology.

[10] Schwarz UP (1998). Preparation of ^211^At-Labeled Humanized Anti-Tac Using ^211^At Produced in Disposable Internal and External Bismuth Targets. Nuclear Medicine and Biology.

[11] Doberenz V (1982). Preparation of astatine of high specific activity in solutions of a given composition. Radiochem. Radioanal. Lett..

[12] Balkin ER (2013). Evaluation of a Wet Chemistry Method for Isolation of Cyclotron Produced [^211^At]Astatine. Applied Sciences.

[13] Yordanov AT (2004). Wet harvesting of no-carrier-added ^211^At from an irradiated ^209^Bi target for radiopharmaceutical applications. Journal of Radioanalytical and Nuclear Chemistry.

[14] Zona C (2008). Wet-chemistry method for the separation of no-carrier-added ^211^At/^211g^Po from ^209^Bi target irradiated by alpha-beam in cyclotron. J Radioanal Nucl Chem.

[15] Neirinckx RD, Smit JA (1973). Separation of astatine-211 from bismuth metal. Analytica Chimica Acta.

[16] Ekberg C, Jensen H, Mezyk SP, Mincher BJ, Skarnemark G (2017). Extraction of ^211^At from nitric acid solutions into various organic solvents for use as an α-source for radiation chemistry studies. J Radioanal Nucl Chem.

[17] Neumann HM (1957). Solvent distribution studies of the chemistry of astatine. Journal of Inorganic and Nuclear Chemistry.

[18] O’Hara MJ (2017). An automated flow system incorporating in-line acid dissolution of bismuth metal from a cyclotron irradiated target assembly for use in the isolation of astatine-211. Applied Radiation and Isotopes.

[19] Bochvarova M, Do KT, Dudova I, Norseev YV, Khalkin VA (1972). Use of columns filled with crystalline tellurium for producing radiochemically pure astatine preparation. Radiokhimiya.

[20] Crawford JR (2017). Development of a preclinical ^211^Rn/^211^At generator system for targeted alpha therapy research with ^211^At. Nuclear Medicine and Biology.

[21] Bennett MR, Maya L, Brown GM, Posey FA (1982). Oxidation of hydroxylamine by nitrous and nitric acids. Inorg. Chem..

[22] Pembridge, J. R. & Stedman, G. Kinetics, mechanism, and stoicheiometry of the oxidation of hydroxylamine by nitric acid. *J. Chem. Soc., Dalton Trans*. 1657–1663, 10.1039/DT9790001657 (1979).

[23] Wilbur DS (2007). Reagents for Astatination of Biomolecules. 2. Conjugation of Anionic Boron Cage Pendant Groups to a Protein Provides a Method for Direct Labeling that is Stable to *in Vivo* Deastatination. Bioconjugate Chem..

[24] Gagnon K (2012). Design and evaluation of an external high-current target for production of ^211^At. Journal of Labelled Compounds and Radiopharmaceuticals.

[25] Li Y (2018). cGMP production of astatine-211-labeled anti-CD45 antibodies for use in allogeneic hematopoietic cell transplantation for treatment of advanced hematopoietic malignancies. PLOS ONE.

