# Supplementary material for: Investigation of a tellurium-packed column for isolation of astatine-211 from irradiated bismuth targets and demonstration of a semi-automated system
Source: Sci Rep. 2019 Nov 18;9:16960. doi: 10.1038/s41598-019-53385-x (PMC6861229; doi:10.1038/s41598-019-53385-x)
Supplement: Supplementary file 1 — Investigation of a tellurium-packed column for isolation of astatine-211 from irradiated bismuth targets and demonstration of a semi-automated system [file 41598_2019_53385_MOESM1_ESM.docx]

**Investigation of a tellurium-packed column for isolation of astatine-211 from irradiated bismuth targets and demonstration of a semi-automated system**

Yawen Li*, Donald K. Hamlin, Ming-Kuan Chyan, Taylor M. Morscheck, Maryline G. Ferrier, Roger Wong, D. Scott Wilbur

Department of Radiation Oncology, University of Washington, Seattle, Washington, United States of America

**Supplementary Information**

**Figure S1.** Our prototype semi-automated system used for ^211^At isolation employing Te-packed columns. The system consisted of a Hamilton Microlab 500 Series Diluter/Dispenser dual-syringe pump and three Modular Valve Positioners (MVPs) controlled by the Microlab 500 Control Software, a dissolution chamber, a Te column, a 250-mL reaction vessel sitting on a magnetic stirrer and a 25-mL loading loop. The gases generated from the HNO_3_ and NH_2_OH·HCl reaction were vented through an ice trap and then a charcoal filter. The entire semi-automated system except for the computer was housed in a two-glove glovebox (Inert, Amesbury, MA) vented through a charcoal filter on the glovebox exhaust and through a secondary charcoal-filtered Plexiglas enclosure placed a radiochemical fume hood.
